# The pollen morphology of *Pelargonium endlicherianum* and *Pelargonium quercetorum* (Geraniaceae) in Turkey

**DOI:** 10.3897/phytokeys.75.11011

**Published:** 2016-12-15

**Authors:** Birol Başer, Mehmet Fırat, Akın Aziret

**Affiliations:** 1 Bitlis Eren University, Faculty of Arts and Science, Department of Biology, Bitlis/Turkey; 2 Yüzüncü Yıl University, Faculty of Education, Department of Biology, Van/Turkey; 3 Fırat University, Keban Vocational School, Department of Environmental Protection, Elazığ/Turkey

**Keywords:** Morphology, Pelargonium, Pollen, Turkey

## Abstract

The pollen morphology of *Pelargonium endlicherianum* Fenzl. and *Pelargonium quercetorum* Agnew. from the family Geraniaceae was examined under light microscopy and scanning electron microscopy. Pollen morphologies are eurypalynous. The pollen grains were tricolporate, prolate-spheroidal and large. Aperture was ectoaperture, colpus was short, and the pores were oblate-spheroidal and large. The exine ornamentations were striate-reticulate and the reticula were heterobrachate. The 2 species are invasive exotics in Turkey.

## Introduction

Geraniaceae is cosmopolitan family of mostly temperate and subtropical annual or perennial herbs and a few small shrubs, comprising about 841 species belonging to 5 genera *Erodium* L’Hérit., *Geranium* L., Monsonia L., *Sarcocaulon* (DC) Sweet, and *Pelargonium* L’Hérit. (Hutchinson 1969, The Plant List 2013). In Turkey, it is represented by 4 (*Biebersteinia* Steph., *Geranium* L., *Erodium* L’Hérit, *Pelargonium* L’Hérit.) genera and 72 taxa in Turkey (Davis 1967). *Pelargonium quercetorum* and *Pelargonium endlicherianum* growing in Turkey are completely natural and uncultivated. These species have a rhizome root structure. The fruit are beaked and they have poured into the surroundings after maturity. The probability of germination is weak. It is usually distributed by rhizomes. It has been reported that *Pelargonium quercetorum* shows distribution only in N. Iraq (Agnew 1967). However, it has been determined that P. Quercetorum grows in a narrow area of Hakkari in Turkey as well. *Pelargonium endlicherianum* species show a natural distribution in eastern and inner Anatolia of Turkey. There are no other *Pelargonium* species close to both of these 2 species. For this reason, there are no ancestor species to hybridize. As a result, there are no hybrids of these 2 species.

The genus *Pelargonium* in the family Geraniaceae numbers over 200 species divided into 15 sections in the world (Van der Walt and Vorster 1981, 1988). It is a familiar group of ornamental plants and represents the sole genus in the tribe Pelargonieae, characterized within the Geraniaceae by zygomorphic flowers and the presence of a hypanthium. The largest concentration of the species occurs in the Cape Province of South Africa, but the genus is also represented in southern central, east, and north-east Africa, Madagascar, western Asia, Australia (Stafford and Gibby 1992). *Pelargonium* is a genus predominantly S. African. Several cultivars of the S. African species are grown as ornamentals in the warmer parts of Turkey. These include *Pelargonium zonale*, *Pelargonium peltatum* and Pelargonium × hybridum (*Pelargonium inquinans* × *Pelargonium zonale*). *Pelargonium endlicherianum* and *Pelargonium quercetorum* from this genus naturally grow in Turkey and N. Iraq. These species are perennial herbaceous or semi-woody bush, have different colored flowers (red, fire red, orange-red, pink, and white edged), and fruit beaked, splitting from base to apex into 5 mericarps (Davis 1967). In a preliminary study of the family Geraniceae, Bortenschlager (1967) demonstrated that, in the compound light microscope, pollen morphology among the different genera was heterogeneous. The genus *Pelargonium* was shown to have some affinities with the related genera *Erodium*, *Monsonia*, and *Sarcocaulon*, and from an examination of 63 species representing all 15 sections of *Pelargonium*, he identified 3 different pollen types: *Pelargonium hymgifolium*, *Pelargonium rapaceum*, and *Pelargonium echinatum*. More recently, the general pollen morphology of the family has been studied by Verhoeven and Marais (1990), who demonstrated that pollen morphological characters were useful in delimiting the different sections of *Pelargonium* and also the subsections of Section Polyactium, although they did not include details of the species investigated or descriptions of the types to aid in identification. Hutchinson (1969) divided the family Geraniaceae into 2 tribus: Geranieae (*Geranium* L., *Erodium* L., *Monsonia* L., and *Sarcocale*) and Pelargonieae (*Pelargonium* L. Herit ex Aiton). Oltmann (1967) defined the pollen morphology of Geraniales. Bortenschlanger (1967) examined 33 *Erodium* L. species and recognized 2 basic types, namely *Geranium multiflorum* and *Erodium* L, and the tectum in all 3 species can be described as striate-reticulate. El-Oqlah (1983) also identified the 2 basic pollen types in *Erodium* L. The shape and size, and apertural type of the grain do not show much variation throughout the pollen type. Pollen type-II is readily distinguished by a reticulatestriate tectum, which is heavily ornamented with baccula and gemmae. Verhoeven and Venter (1987) reported a similar pollen type in *Erodium* L. (except Erodium oxyrrhynchum ssp. oxyrrhyncum striate-reticulate tectum with gemmate and baculate muri). The morphology of the pollen grains of all 3 species corresponds to that of the rest of the genus *Pelargonium*, in that the grains are spherical and tricolporate (Marais 1991). The structure or the wall of the pollen grain is semitectate (Verhoeven and Marais 1990, Marais 1997) *Pelargonium hirtipetalum*, *Pelargonium pubipetalum*, and *Pelargonium aridicola* were described as new species, and they correspond with regard to the leaf anatomy, the structure of the androecium and pollen morphology (Perveen and Gaiser 1999) The pollen morphology of 13 species belonging to the 3 genera of the family Geraniaceae was investigated with a light microscopy (LM) and scanning electron microscopy (SEM). The pollen morphology of Geraniaceae or some of its representatives have been studied by several researchers (Kuprianova and Alyoshina 1972, Moore and Webb 1978, El-Oqlah 1989, Stafford and Blackmore 1991). Recentely, Aedo et al. (2007) and Shehata (2008) have used the palynological data in their taxonomic revision of the genera *Pelargonium* and *Geranium*. Boukhris et al. (2013) performed a study of the essential oil composition, and trichomes distribution, morphology, and anatomy of the aerial organs of *Pelargonium graveolens* L., which is an aromatic and medicinal species originating from Sfax (Tunisia)

The systematic investigation of the pollen morphology of *Pelargonium* in Turkey has not been studied comprehensively. According to Flora of Turkey, *Pelargonium quercetorum* differs from *Pelargonium endlicherianum* by its larger size, less hairy or glabrescent leaves with lobed and dentate segments, more numerous shorter pedicels, and narrower upper petals (Davis 1967). These 2 species are systematically different from each other. The purpose of this study was to determine pollen morphological differences in *Pelargonium* of Turkey and to take these differences into account.

The terminology of pollen morphology was used based on Erdtman (1952), Kremp (1965), Faegri and Iversen (1964), Walker and Doyle (1976), and Punt et al. (1994).

## Materials and methods

While *Pelargonium endlicherianum* was collected from Hakkari in the C9 grid of Flora of Turkey, *Pelargonium quercetorum* was collected from Tunceli in the B7 grid of Flora of Turkey. The pollen characteristics of these 2 species were examined in preparations by the method of Woodhouse (1935) for the LM. The polar and equatorial axis, colpus length and width, and exine and intine thickness of the pollen were measured 30 times. The morphological characteristics under the LM were measured using an Olympus BX41 microscope. Microphotographs were taken from this microscope’s camera. During SEM, selected dry samples of pollens were placed on the aluminum stabs with the help of double-sided adhesive tape and coated in gold with a vacuum. The images of the pollen were taken with a Jeol JSM 7001-F SEM in the SEM laboratory of the Department of Biology, Fırat University.

## Results

**Figure 1.**
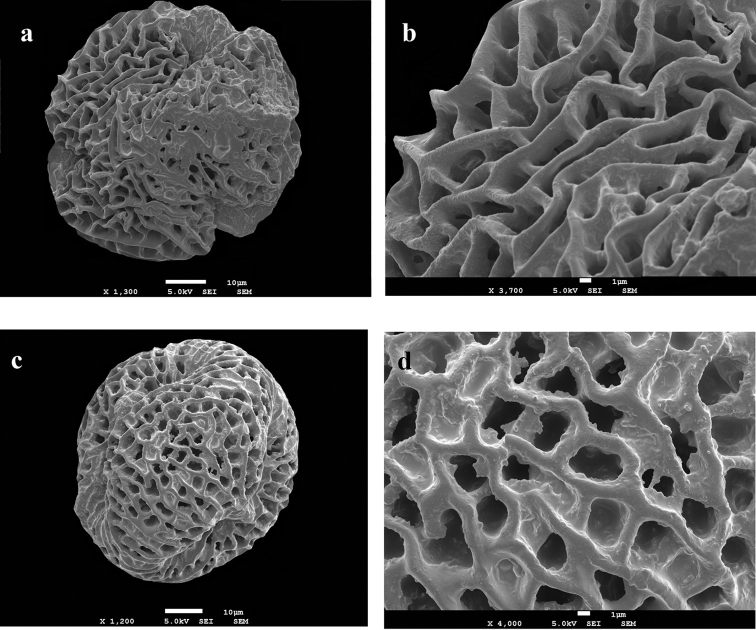
Light microscope microphotography; *Pelargonium endlicherianum*: **a** Polar sight **b** Ekvatoryal sight **c** exine ornamentation, *Pelargonium quercetorum* **d** Polar view **e** Equatorial view **f** exine ornamentations. Scale: **a–f** = 20 µm.

**Figure 2.**
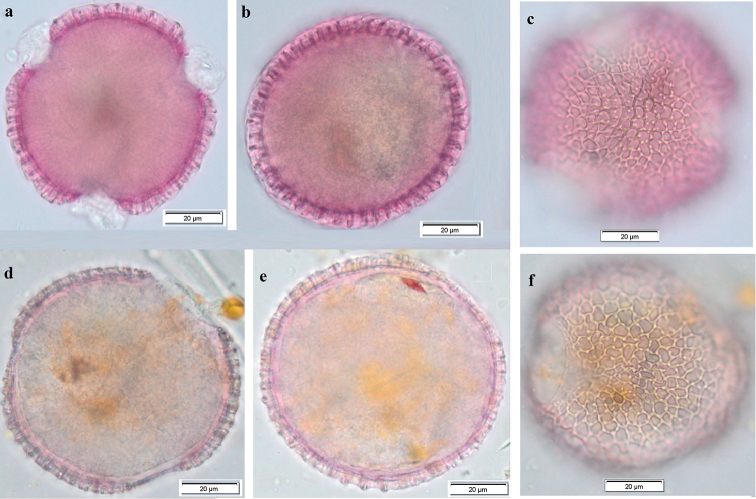
SEM microphotography; *Pelargonium endlicherianum*: **A** Polar view (×1,300) **B** exine surface (×3,700). *Pelargonium quercetorum*: **c** Equatorial view (×1,200) **d** exine surface (×4,000).

The morphological variation of the pollen grains of 2 species of *Pelargonium* was described in terms of the size and shape of the pollen grains, morphology of apertures, and exine ornamentation. Specifically, the surface ornaments were defined in detail using the SEM microphotographs. Tables 1 and 2 show the pollen size variations and measurements. Figs 1–2 illustrate the representative pollen characters.

**Table 1.** The measurements of *Pelargonium endlicherianum* vs *Pelargonium quercetorum* in ligth microscope.

Taxon	Polar axis min-max (µm)	Equatorial axis min-max (µm)	P/E ratio (polen shape)	Colpus length min-max (µm)	Colpus width min-max (µm)	Por length min-max (µm)	Por width min-max (µm)	Exine thickness min-max (µm)	Intine thickness min-max (µm)
*Pelargonium endlicherianum*	85.93±6.69 75–100	89.30±7.13 77–109	0.96	50.37±3.47 44–57	24.78±2.89 20–29	23.01±2.89 19–32	21.93±2.27 18–26	5.40±0.44 4.75–6	1.84±0.26 1.5–2.25
*Pelargonium quercetorum*	81.53±4.45 73–92	79.33±2.87 75–87	1.03	47.60±3.93 40–55	16.93±2.60 13–23	21.40±2.37 17–25	17.20±2.02 12–21	4.80±0.57 4–5.50	1.57±0.35 1–2

### The morphological characteristics of the pollen

#### Pelargonium endlicherianum

Taxon classificationPlantaeGeranialesGeraniaceae

##### Pollen shape.

Tricolporate

##### P/E ratio.

0.96

##### Shape.

prolate-spheroidale.

##### Aperture.

Ectoapertur and colpus are short, pore shape oblate-spheroidale and large.

##### Ornamentation.

Striate-reticulate.

##### LM measurements.

Polar axis (P) 73.00–81.53–92.00 µm, equatorial axis (E) 77.00–89.30–109.00 µm, P/E ratio: 0.96, colpus length 44.00–50.37–57.00) µm, Pore length 19.00–23.01–32.00 µm, exine thickness 4.75–5.40–6.00 µm, intine thickness 1.50–1.84–2.25 µm (Table 1, Fig. 1a, b).

##### SEM measurements.

The number of lumina per 10 µm^2^ is 5–6, the size of the lumina of the pollen grains is approximately between 2.42 µm to 5.71 µm, and the average thickness of the muri is 0.62 µm to 0.88 µm, polar axis (P): 79 µm, equatorial diameter (E) 85 µm (Fig. 2).

#### Pelargonium quercetorum

Taxon classificationPlantaeGeranialesGeraniaceae

##### Pollen shape.

Tricolporate

##### P/E ratio.

1.03

##### Shape.

Prolate-spheroidal.

##### Aperture.

Ectoaperture and colpus are short, pore shape prolate-sphreroidal and large.

##### Ornamentation.

Striate-reticulate.

##### LM measurements.

Polar axis (P) 73.00–81.53–92.00 µm, equatorial axis (E) 75.00–79.33–87.00 µm, P/E orani: 1.03, colpus length 40.00–47.60–55.00) µm, Pore length 17.00–21.40–25.00 µm, exine thickness 4.00–4.80–5.50 µm, intine thickness 1.00–1.57–2.00 µm (Table 1, Fig. 1c–d).

##### SEM measurements.

The number of lumina per 10 µm^2^ is 4–5, the size of the lumina of the pollen grains is approximately between 2.50 µm to 5.63 µm, and the average thickness of the muri is 1 µm to 1,25 µm, polar axis (P): 69 µm, equatorial diameter (E) 67 µm (Fig. 2).

## Discussion

The present study aimed to: 1) survey the pollen morphology of 2 taxa belonging to *Pelargonium* growing naturally in Turkey, and 2) to determine pollen morphological differences of this genus. The first comprehensive report on the pollen morphology of the 2 taxa belonging to the genus *Pelargonium* was examined using LM and SEM. The pollen morphology of Geraniaceae is eurypalynosis; The pollen morphology of Geraniaceae is significant in the systematics of the genus. The pollens are usually radial symmetric, isopolare, oblate-spheroidal, rarely sub-oblate, tricolporate, rarely colpate, colpuses are short, and the sexine is thicker than the nexine. Tectum dense-reticulate. baculate or gemmate muri or striate (Erdtman 1952, 1966, 1969). The pollen grains of *Pelargonium* are usually trizonocolporate. The size of the grains varies from P 47–120 µm E 47–120 µm. The pollen grains are characterized by a distinctive reticulum, which may be multi-layered with striate elements of various lengths and thicknesses. Ornamentations; reticulate, coarsely-reticulate, striate/reticulate (Stafford and Gibby 1992). The species examined in this study were separated into 4 main pollen types and 5 subgroups, summarized as follows: Type 1: *Pelargonium longifolium*, Type 2: *Pelargonium echinatum*, Type 3: *Pelargonium hirtum*, and Type 4: *Pelargonium rapaceum* type (Subgroup A: *Pelargonium tragacanthoides*, Subgroup B: *Pelargonium fasciculaceum*, Subgroup C: *Pelargonium schlechteri*). In their work, the species *Pelargonium endlicherianum* was measured and recorded as P: 94–(96.6)–100 µm, E: 83–(91)–95 µm and exine: 7–9 µm, pollen shape prolate-spheroidal, ornamentation striate/reticulate and was identified as type 3, *Pelargonium hirtum*. Based on our study, we had the following results, which are consistent with the previous work: *Pelargonium endlicherianum* (P: 75–(85.93)–100 µm, E: 77–(89.30)–109 µm, exine: 5.40 µm), striate/reticulate, and pollen shape; prolate-spheroidal. The results of the micromorphical investigations we conducted on the pollens of the other studied species, which were *Pelargonium quercetorum* (P: 73.00–81.53–92.00 µm, E: 75.00–79.33–87.00 µm exine: 4.80 µm and pollen shape; prolate-spheroidal), and these were understood to be in accordance with their evaluations and were accepted as type 3. In their work, species *Pelargonium endlicherianum* were P: 94–(96.6)–100 µm, E: 83–(91)–95 µm and exine: 7–9 µm, pollen shape prolate-spheroidal, ornamentation striate/reticulate and type 3. The classification of *Pelargonium* pollen types in terms of ornamentation and exine structure does not clearly conform to the generic boundaries within the family Geraniaceae or sectional divisions within the genus. The most frequent pollen type in the genus *Pelargonium* is Pelargonium hirtum, which has been found in species from every section of the genus. In another study on the family of Geraniaceae, The studies on pollen morphology and taxonomic importance of the family Geraniaceae in Egypt have shown the existence of 3 main types and 3 subtypes in terms of the aperture type, exine structure, exine surface, and pollen type (P/E ratio): Type I: striate/striate-reticulate, Type II: reticulate/gemmate type, and Type III: reticulate type (Subtype A: *Monsonia heliotropioides*, Subtype B: *Monsonia senegalensis*, and Subtype C: *Pelargonium grandiflorum*). It was determined that there was a distinctive striation on the tricolpate and ornament of the species *Pelargonium grandiflorum*, which is a type of *Pelargonium* (Shehata 2008). It was determined in the present study that the pollen sizes, apertures, and ornamentations of the species *Pelargonium endlicherianum* and *Pelargonium quercetorum* showed differences compared to the pollens of *Pelargonium grandiflorum* studied by Shehata (2008) (Table 2, Fig. 2). The pollen was examined and separated into pollen types representing the lowest recognizable taxonomic units on the basis of the pollen morphological characteristics, principally, the exine structure and ornamentation. The greater majority of species were found to fall into a general striate/reticulate ornamentation type within which it was not possible to distinguish between species, although a number of other pollen types and subgroups could be identified by differences in their reticulum and ornamentation, the palynological results are discussed with respect to current systematic treatments of *Pelargonium* (Stafford and Gibby 1992).

**Table 2.** The comparison of species *Pelargonium endlicherianum*, *Pelargonium quercetorum*, and *Pelargonium grandiflorum*.

Pollen	*Pelargonium endlicherianum*	*Pelargonium quercetorum*	*Pelargonium grandiflorum* (Shehata A. A, 2008)
**Polar axis**	85.93 (75–100) µm	81.53 (73–92) µm	44 (42–46) µm
**Equatorial axis**	89.30 (77–109) µm	79.33 (75–87) µm	32 (36–38) µm
**Shape**	Prolate-spheroidal	Prolate-spheroidal	Prolate
**Ornamentations**	Striate-reticulate	Striate-reticulate	Reticulate
**Aperture**	Tricolporate	Tricolporate	Tricolpate

In conclusion, we found a correlation between our results and the classification of the taxa in this genus, that is, pollen features, especially pollen size, exine and ornamentation, proved to be the most useful characters for the systematics of the taxa.

## Supplementary Material

XML Treatment for Pelargonium endlicherianum

XML Treatment for Pelargonium quercetorum
